# Supplementary material for: Content-rich biological network constructed by mining PubMed abstracts
Source: BMC Bioinformatics. 2004 Oct 8;5:147. doi: 10.1186/1471-2105-5-147 (PMC528731; doi:10.1186/1471-2105-5-147)
Supplement: Additional File 2 — The original results of the above study (non-essential files are deleted to keep the file size under the limit set by BMC bioinformatics). [file 1471-2105-5-147-S2.bz2 › chilibotAdditionalFile2/dip10/738ID11495998E18104/html/GCVA_GCVR.html]

 


 **GCVA** and **GCVR** 
  
Found 10 abstracts in PubMed, retrieved 10.  
 

 What does Google say? 
 PDF only 
| .edu only 

---

**Interactive relationship** (e.g. stimulation, inhibition, etc)

**Neutral relationship**- Transcription of the gcv operon is positively regulated by  **GcvA**  and negatively regulated by  **GcvA**  and  **GcvR** .  Ref: 11495998 Microbiology, 2001
- Glycine binds the transcriptional accessory protein  **GcvR**  to disrupt a  **GcvA**   **GcvR**  interaction and allow  **GcvA**  mediated activation of the Escherichia coli gcvTHP operon.  Ref: 12101307 Microbiology, 2002
- Using a LexA based system for analysing protein heterodimerization, it is shown that  **GcvR**  interacts directly with  **GcvA**  in vivo to repress gcvTHP expression.  Ref: 11495998 Microbiology, 2001
- Negative regulation of a gcvT lacZ fusion by  **GcvR**  was shown to require  **GcvA** , a LysR family protein known to both activate gcv in the presence of glycine and repress gcv in the presence of purines.  Ref: 7665475 J Bacteriol, 1995
- Transcription from the gcvB promoter is activated by the  **GcvA**  protein and repressed by the  **GcvR**  protein, the transcriptional regulators of the gcvTHP operon encoding the enzymes of the glycine cleavage system.  Ref: 10972807 Mol Microbiol, 2000
- Rather, glycine binds to  **GcvR** , disrupting a  **GcvA**   **GcvR**  interaction required for repression and allowing  **GcvA**  activation of the gcvTHP operon.  Ref: 12101307 Microbiology, 2002
- Repression by  **GcvA**  is dependent on a second regulatory protein,  **GcvR** .  Ref: 12101307 Microbiology, 2002
- Finally, it is shown that the C terminal half of  **GcvA**  is involved in its interaction with  **GcvR** , whilst the entire  **GcvR**  protein appears to be necessary for heterodimerization.  Ref: 11495998 Microbiology, 2001
- However, overexpression of  **gcvR**  re established purine mediated repression of the gcvT lacZ fusion, supporting the model for gcv regulation that suggests the need for a relatively constant  **GcvA**  to  **GcvR**  ratio for appropriate regulation of gcv expression in response to glycine and purine availability.  Ref: 10463182 Microbiology, 1999
- **GcvR**  interacts with  **GcvA**  to inhibit activation of the Escherichia coli glycine cleavage operon.  Ref: 11495998 Microbiology, 2001

**Non-interactive relationship** (e.g. studied together, co-existance, homology, etc.)

- The Escherichia coli gcv operon is transcriptionally regulated by the  **GcvA** ,  **GcvR** , Lrp, and PurR proteins.  Ref: 10074087 J Bacteriol, 1999
- To determine the role of site 1 in regulation, single and multiple base changes were made in site 1 and tested for their ability to alter  **GcvA**  mediated activation and  **GcvA**   **GcvR**  mediated repression.  Ref: 11065369 Microbiology, 2000
- Models explaining how  **GcvR**  and  **GcvA**  might interact to regulate gcv expression are proposed.  Ref: 7665475 J Bacteriol, 1995
- In addition, it was shown that  **gcvR**  lacZ expression is neither regulated by the glycine cleavage activator protein  **GcvA**  nor autogenously regulated by  **GcvR** .  Ref: 9537378 J Bacteriol, 1998
- Activation was also dependent on the  **GcvA**  protein, and repression was dependent on both the  **GcvA**  and  **GcvR**  proteins, demonstrating that the roles for these proteins were not altered.  Ref: 10217490 Microbiology, 1999
- Several mutations in either  **gcvA**  or  **gcvR**  that result in a loss of gcv repression also result in a loss of  **GcvA**   **GcvR**  heterodimerization.  Ref: 11495998 Microbiology, 2001
